# The Human Sodium-Glucose Cotransporter (hSGLT1) Is a Disulfide-Bridged Homodimer with a Re-Entrant C-Terminal Loop

**DOI:** 10.1371/journal.pone.0154589

**Published:** 2016-05-03

**Authors:** Louis J. Sasseville, Michael Morin, Michael J. Coady, Rikard Blunck, Jean-Yves Lapointe

**Affiliations:** Groupe d'étude des protéines membranaires (GÉPROM) and Département de physique, Université de Montréal, Montréal, Québec; Ecole Polytechnique Federale de Lausanne, SWITZERLAND

## Abstract

Na-coupled cotransporters are proteins that use the trans-membrane electrochemical gradient of Na to activate the transport of a second solute. The sodium-glucose cotransporter 1 (SGLT1) constitutes a well-studied prototype of this transport mechanism but essential molecular characteristics, namely its quaternary structure and the exact arrangement of the C-terminal transmembrane segments, are still debated. After expression in *Xenopus* oocytes, human SGLT1 molecules (hSGLT1) were labelled on an externally accessible cysteine residue with a thiol-reactive fluorophore (tetramethylrhodamine-C5-maleimide, TMR). Addition of dipicrylamine (DPA, a negatively-charged amphiphatic fluorescence “quencher”) to the fluorescently-labelled oocytes is used to quench the fluorescence originating from hSGLT1 in a voltage-dependent manner. Using this arrangement with a cysteine residue introduced at position 624 in the loop between transmembrane segments 12 and 13, the voltage-dependent fluorescence signal clearly indicated that this portion of the 12–13 loop is located on the external side of the membrane. As the 12–13 loop begins on the intracellular side of the membrane, this suggests that the 12–13 loop is re-entrant. Using fluorescence resonance energy transfer (FRET), we observed that different hSGLT1 molecules are within molecular distances from each other suggesting a multimeric complex arrangement. In agreement with this conclusion, a western blot analysis showed that hSGLT1 migrates as either a monomer or a dimer in reducing and non-reducing conditions, respectively. A systematic mutational study of endogenous cysteine residues in hSGLT1 showed that a disulfide bridge is formed between the C355 residues of two neighbouring hSGLT1 molecules. It is concluded that, 1) hSGLT1 is expressed as a disulfide bridged homodimer via C355 and that 2) a portion of the intracellular 12–13 loop is re-entrant and readily accessible from the extracellular milieu.

## Introduction

Ion-coupled membrane cotransporters are molecular machines that use the electrochemical energy of a transmembrane ionic gradient to energize the transport of another solute. While the general concept of alternating-access mechanism has been put forward as a general *modus operandi* for a large number of cotransporters [1], understanding this mechanism at the atomic level is still very much in progress.

The sodium glucose cotransporter 1 (SGLT1) has been the subject of extensive structure/function studies using a variety of experimental approaches [2–9]. Based on the analysis of steady state and pre-steady state cotransport currents, a reliable 7-state kinetic model was proposed [10]. While the kinetics of cotransport is now better understood, other important characteristics remain to be experimentally established. Two of these characteristics will be addressed in the present study: 1) the multimeric state of SGLT1 expressed in the membrane and, 2) the membrane topology of the long loop present between transmembrane segments 12 and 13 [11] (numbering according to the LeuT nomenclature, where the first N-terminal transmembrane segment (TM) of SGLT1 becomes TM -1, followed by TM1, and so on). The availability of crystal structures for a bacterial homolog of SGLT1 (the V*ibrio parahaemolyticus* sodium-galactose transporter, vSGLT) [12, 13] did not help in solving the issues of the oligomeric state. As a monodisperse solutions containing only a single oligomeric state is required for crystallisation [14], a given multimeric state in a crystal does not imply that the same state is kept in a membrane environment [15]. Also regarding the membrane topology of the 12–13 loop, the crystal structures of vSGLT did not provide a final response. Although in the vSGLT crystals, the C-terminal end of the TM12 and the N-terminal end of TM13 were both located on the intracellular side of the protein, sequence alignments suggest no recognisable homology between the 12–13 loop of human SGLT1 (hSGLT1) which is 90 residues long and that of vSGLT which is ~24 residue long. In fact, very little homology exists downstream of TM12 between the 12 members of the SLC5A family of cotransporters. Previous reports suggested that a portion of the loop in SGLT1 was accessible from the extracellular space [8, 16, 17].

In the present study, we used hybrid voltage sensors (hVoS [18]) and fluorescence resonance energy transfer (FRET) to examine these issues. hSGLT1 will be labelled with maleimide-linked fluorophores on accessible cysteine residues already present or introduced in the cotransporter through mutation. The wt hSGLT1 has 15 cysteine residues but none of them can be labelled from the extracellular solution [3]. Fluorescence intensities will be studied in voltage-clamp conditions in the presence of dipicrylamine (DPA), an amphiphatic anion, which can act as an energy acceptor from TMR or Alexa-488. Due to its negative net charge, DPA distributes between the two membrane leaflets according to the membrane potential. Depolarizing pulses produced a voltage-dependent fluorescence signal which has to come exclusively from fluorophores that are within ~40–60 Ǻ from a DPA molecule located in the lipid membrane. This provides a powerful tool for establishing the position of a fluorophore with respect to the membrane plane (i.e. on the intracellular or extracellular side of the membrane) and allow for an unambiguous detection of energy transfer between two neighbouring cotransporter molecules.

## Materials and Methods

### DPA quenching and hVoS FRET quenching

Being an amphipathic anion, DPA distributes between the two leaflets of the membrane in a voltage-dependent manner [19, 20]. By depolarising or hyperpolarising the membrane, we can accumulate DPA on the intra- or on the extracellular membrane leaflets, respectively. According to previous electrophysiological experiments in oocytes, DPA would be completely on the extracellular side at -150 mV, equally distributed at -50 mV and completely on the intracellular side at +50 mV [19].

**Fig 1:**
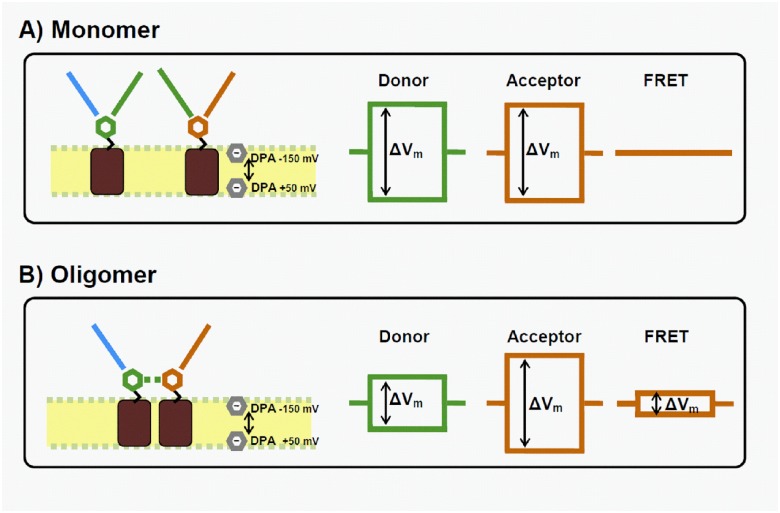
Principle of hVoS FRET quenching. Dipicrylamine (DPA, grey hexagon) distribution on either side of the membrane is controlled via the membrane potential (V_m_). Changes in V_m_ (ΔV_m_) thus reduce/increase the mean distance between DPA and fluorescent probe (Alexa 488, AL488, as green hexagon and Tetramethylrhodamine, TMR, as orange hexagon). Two possibilities are considered. A) If only monomers are present, FRET between AL488 and TMR does not occur. Thus, only DPA quenching of AL488 (using the donor filter set) and TMR (using the acceptor filter set) is observed, but no fluorescence signal is detected using the FRET filter set. B) If multimeric states are present, FRET occurs between AL488 and TMR. A voltage-dependent fluorescent signal can be seen using the 3 filter sets.

Due to its absorption in the visible range, it acts as acceptor for FRET experiments from the SGLT-linked fluorophores. DPA’s voltage-dependent displacement will affect the fluorescence intensity of a fluorophore depending on its position with respect to the membrane plane. A fluorophore located toward the extracellular face of the membrane will emit higher fluorescence intensity upon depolarisation as DPA is moving inside (see Fig 1). In contrast, a fluorophore located towards the intracellular side will emit lower fluorescence intensity upon depolarisation. Thus, in comparison with previous fluorescence studies where only the accessibility of a given residue was analyzed, utilisation of DPA allows for a clear determination of intra- or extracellular position of labeled residues, depending on the unambiguous effect of membrane potential on the observable fluorescence intensity.

A previous study from our group showed that cysteine residues at position 255 and 511 (C255 and C511) formed a disulfide bridge in hSGLT1 [4]. Accordingly, in the mutant C255A, the sulfur group of the partner cysteine residue (C511) becomes available and can be labelled with tetramethylrhodamine-C5-maleimide (TMR) or Alexa 488-C5-maleimide (AL488). When two appropriate fluorophores (a fluorescence donor and a fluorescence acceptor) are near each other, the excited donor can transfer its energy to the acceptor through FRET (see Fig 1B). The calculated Förster distance (R_0_) of AL488/TMR is 59 Å, which is appropriate to detect the presence of multimeric form of hSGLT1 as the diameter of a vSGLT monomer is ~ 40 Å. In the present study, we will detect a FRET signal between TMR-labelled and AL488-labelled hSGLT1 in the presence of dipicrylamine (DPA). Coupled with fluorescent molecules (TMR and AL488, in our case) this system, sometimes dubbed hybrid Voltage Sensor (hVoS), has been shown to greatly increase the sensibility of fluorescent studies [18–20]. The voltage-dependent effect of DPA will have a large effect on the fluorescence level recorded with a FRET filter set as DPA with its large absorption spectrum will quench both the donor and the acceptor. The calculated R_0_’s are 55 and 35 Å for the AL488/DPA and TMR/DPA pairs, respectively. By using DPA, the FRET signal coming exclusively from membrane-bound TMR and AL488 becomes voltage-dependent (hVoS FRET quenching) and easily detectable (see Fig 1).

### Oocyte Preparation and Injection

Oocytes were surgically removed from *Xenopus laevis* frogs and defolliculated as previously described [21]. All frog manipulations (anaesthesia, surgery and sacrifice of the frogs after the final collection of oocytes) were performed in accordance with the Canadian guidelines and have been specifically approved by the ethics committee (CDEA, protocol #15–042) of the Université de Montréal. Immersion in a bath containing 1.3 g/l of tricaine was used to anaesthetise the frog before surgery. A 3 month period was observed between successive surgeries on any given frog and after 2 or 3 surgeries the frogs were sacrificed by a prolonged immersion in the same anesthetic bath. One day after defolliculation, oocytes were injected with 46 nl of mRNA solution (0.1 and 0.25 μg/μl for wt hSGLT1 and mutants) in order to obtain maximal protein expression. Oocytes were maintained in Barth's solution (in mM: 90 NaCl, 3 KCl, 0.82 MgSO_4_, 0.41 CaCl_2_, 0.33 Ca(NO_3_)_2_, 5 HEPES, pH 7.6) supplemented with 5% horse serum, 2.5 mM Na^+^-pyruvate, 100 U/ml penicillin, and 0.1 mg/ml streptomycin for 4–5 d before being used in an experiment.

### Voltage-clamp fluorometry

In brief, oocytes were preincubated on ice for 10 min before the saline solution was exchanged with a solution containing either 0.25 μM TMR, 2 μM AL488 or 0.25 μM TMR + 2 μM AL488 (all maleimide-based fluorophores). After a 10 min labelling period, oocytes were washed twice and kept on ice until used.

Measurements were performed with the cut-open voltage-clamp fluorometry technique for spatial voltage homogeneity and fast temporal resolution [22]. Fluorescence was measured at the animal pole. Macroscopic currents were recorded using GPatch acquisition software (Department of Anesthesiology, University of California, Los Angeles, Los Angeles, CA). Oocytes were placed in a three-compartment chamber, top, middle, and bottom, containing an external solution (90 mM NaCl, 3 mM KCl, 0.82 mM MgCl_2_, 0.74 mM CaCl_2_, 10 mM Hepes, adjusted to pH 7.6 using TRIS) and then permeabilized by adding 0.2% saponin to the bottom chamber solution. After less than 3 minutes, the presence of saponin has decreased the access resistance to the oocyte cytosol as monitored by the capacitance and the bottom solution was exchanged with an internal solution (5 mM NaCl, 25 mM KCl, 65 mM K-Gluconate, 10 mM Hepes, 1 mM EGTA, adjusted to pH 7.6 using TRIS). A micropipette filled with 3 M KCl and inserted into the oocyte was used to measure the electrical potential across the membrane patch exposed to the top chamber. As wt hSGLT1 has been shown to be resistant to labelling with maleimide-based fluorophore [3], wt hSGLT1-expressing oocytes were used as controls, effectively allowing characterisation of the nonspecific fluorescence labeling.

The oocytes were clamped to a resting membrane potential (V_m_) of -50 mV, and 5 repetitions of V_m_ steps to +50 and -150 mV, 300 ms in duration, were applied with an interval of 1.7 s between each pulse. The 5 repetitions were averaged online for each experiment. For all experiments, the fluorescence level during voltage step was first monitored without DPA using appropriates filter set, DPA was added to the top chamber (reaching a concentration of ≈ 2.5 μM), and fluorescence was again monitored through voltage steps. Three filter sets were used. For AL488, a 470 ± 20 nm excitation filter (Ex), a 495 nm-longpass dichroic mirror (Di) and a 525 ± 25 nm emission filter (Em) were used. For TMR, we used: Ex.: 540 ± 10 nm; Di: 550 nm LP; Em: 605 ± 70 nm. For detecting FRET we used: Ex: 470 ± 20 nm; Di: 495 nm LP; Em: 605 ± 27.5 nm, (all from Chroma Technologies, VT, USA).

### Western blots

Western blots were performed as previously described [21] using a total membrane preparation obtained from oocytes expressing a myc-tagged hSGLT1 or aquaporin 2 (AQP2). Samples obtained from 1 to 4 oocytes were run on a 7.5 or a 10% polyacrylamide gel and transferred onto a nitrocellulose membrane. The efficiency of the overall procedure was monitored by Ponceau red staining. To prevent non-specific binding of antibodies, the membranes were first blocked with 5% non-fat milk in TBS-T (Tris-buffered saline + Tween 20, 0.1%) and then probed with the specific antibody (α-c-myc 1:500, Enzo Life Science, ON, CA or α-AQP2 (N-20) 1:500, Santa Cruz Biotech, CA, USA) followed by incubation with secondary antibody (HRP-linked chicken anti-mouse 1:25,000, Santa Cruz Biotech or HRP-linked chicken anti-goat 1:25,000, Santa Cruz Biotech). Blots were revealed using enhanced chemiluminescence detection (Phototope-HRP, New England Biolabs, Pickering, ON, Canada).

The reducing agent β-Mercaptoethanol (β-ME) is specifically used during Western blotting to break disulfide bridges. As it is possible that inter-subunit disulfide bridges contribute in holding a multimeric hSGLT1 complex, we performed Western blots in the presence and in the absence of β-ME to see if breaking disulfide bridges would affect the migration profile of hSGLT1. Also, mutants where cysteines were replaced by alanines were tested with/without β-ME to assess their involvement of specific cysteine residues in a putative disulfide bridge holding a multimeric state together.

### Data analysis

Unless otherwise described, all data are expressed as means ± SE. Statistical analysis were performed with Student’s t-test unless otherwise mentioned.

## Results

### Molecular proximity between SGLT1 molecules

**Fig 2:**
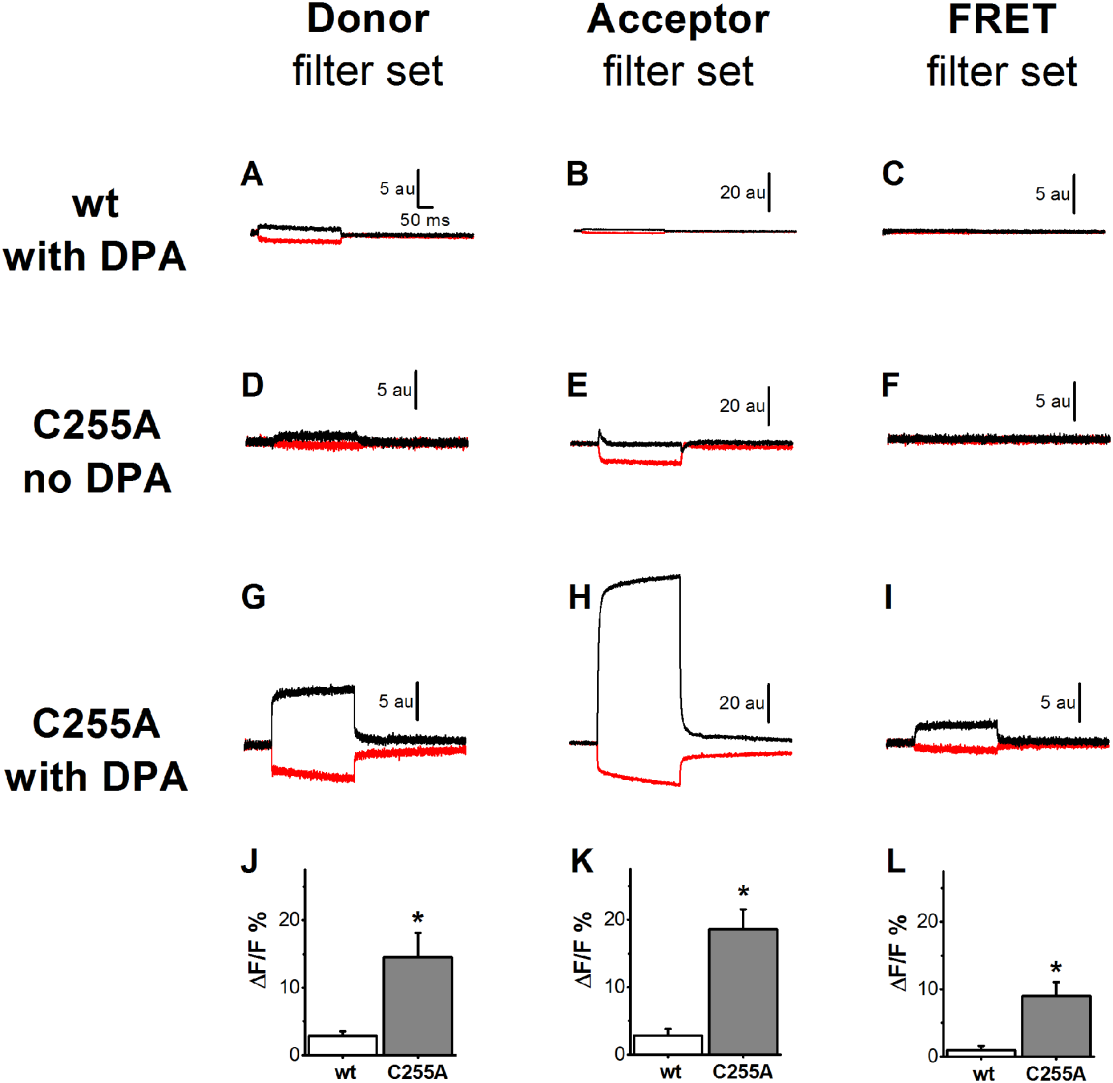
hVoS FRET quenching results. Typical fluorescence traces for oocytes expressing either wt hSGLT1 (panel A through C) or the hSGLT1 mutant C255A (panel D through I) which is known from a previous study [3] to present a cystein residue (C511) that is freely accessible for labelling from the external solution. Oocytes are simultaneously labeled with Alexa 488 (AL488, the donor) and Tetramethylrhodamine (TMR, the acceptor). In the absence of dipicrylamine (DPA, a negatively-charged amphiphatic fluorescence quencher), changing the membrane potential from -50 mV to +50 mV (black traces) or to -150 mV (red traces) produces very little change in the fluorescence intensities measured for C255A expressing oocytes using either one of the 3 filter sets (panel D to F). In the presence of DPA, oocytes expressing C255A present strong voltage-dependent fluorescent signals (panel G to I). This effect is specific for the fluorophores attached to C511 as wtSGLT1 (panel A to C) display much weaker voltage-dependent fluorescent signal. Panels J to L compare the mean voltage-dependent signal $\frac{F_{+50mV}-F_{-150mV}}{F_{-50mV}}$ (mean ± SE) using the 3 filter sets for oocytes expressing wt SGLT1 (n = 5) or the mutant C255A (n = 15 to 17). Stars (*) denote statistical significance (C255A vs wt, p<0.05).

hVoS FRET quenching experiments were performed to determine whether C255A forms a monomer or a multimeric complex in the membrane. As described, wt and C255A-expressing oocytes were labeled using a solution containing 2 μM Alexa 488-C5-maleimide (AL488) and 0.25 μM tetramethylrhodamine-C5-maleimide (TMR). As shown in Fig 2, this provides a high level of TMR fluorescence as compared to AL488 which maximises the probability that the fluorescence donor (AL488) has an acceptor (TMR) if hSGLT1 is functioning in a multimeric configuration.

At a holding potential of -50 mV, we first measured how the addition of DPA to the extracellular solution affected the fluorescence levels in order to estimate the fraction of AL488 and TMR within the FRET-range of membrane-bound DPA. In the case of control oocytes expressing wt hSGLT1 and exposed to AL488 and TMR, the addition of DPA produced a decrease in fluorescence intensity by 28 ± 4% for AL488 and by 11 ± 3% for TMR (n = 5 oocytes). The larger energy transfer efficiency of AL488-DPA is consistent with the fact that R_0_ is larger for AL488-DPA (55 Å) than for TMR-DPA (35 Å), leading to less fluorophores within the FRET-range in the latter case. In the case of oocytes expressing C255A, the fluorescence intensities decreased by 27 ± 4% and 25 ± 3% for AL488 and TMR, respectively (n = 5, in each case). This suggests that for AL488, specifically bound and non-specifically bound fluorophores are both within the reach of DPA. In case of TMR, due to the shorter R_0_ associated to the TMR/DPA pair, the fluorophores that are specifically bound to C255A are more readily accessible to DPA than the fluorophores that are non-specifically bound to the membrane.

Following addition of DPA to the top bath chamber, the effect of V_m_ steps from a holding potential of -50 mV to +50 mV and -150 mV was assessed. In the case of oocytes expressing C255A, the presence of DPA produces a clear voltage-dependent quenching of TMR and AL488 fluorescence (see Fig 2G and 2H in the presence of DPA compared to Fig 2D and 2E in the absence of DPA). In the case of control oocytes expressing wt hSGLT1, the effect of DPA at +50 and -150 mV is 5 to 7 times smaller than for C255A-expressing oocytes (compare Fig 2A and 2B with Fig 2G and 2H). In average, V_m_ steps produce change in AL488 fluorescence by 14.5 ± 3.6% ($\frac{F_{+50mV}-F_{-150mV}}{F_{-50mV}}$, n = 14) and 2.8 ± 0.7% (n = 5) for C255A and wt, respectively (Fig 2J). In the case of TMR, going from +50 to -150 mV produces a change in fluorescence by 18.5 ± 3.0% (n = 16) and 2.8 ± 1.0% (n = 5) for C255A and wt, respectively (Fig 2K). In contrast, in the absence of DPA, for oocytes expressing C255A and labelled with AL488 and TMR, the effect of V_m_ steps averaged 3.0 ± 0.7% and 0.9 ± 0.4% for AL488 (n = 8) and TMR (n = 7), respectively (Fig 2D and 2E for a representative example). Taken together, this shows that a dominant portion of the voltage-dependent fluorescence signal is coming from the effect of DPA on the fluorophores that are specifically attached to the exposed cysteine residue of mutant C255A.

Important information can also be obtained from the directionality of the fluorescence intensity change following a voltage step in the presence of DPA [20]. Presence of a disulfide bridge was previously shown between C255 and C511 [3, 4]. Thus, in the C255A cotransporter, C511 is available for labelling with a thiol-reactive fluorophore. Sequence alignment between hSGLT1 and vSGLT puts C511 on the 11–12 extracellular loop [12, 23]. This is clearly confirmed by the fact that moving DPA to the extracellular leaflet by hyperpolarisation to -150 mV reduces the fluorescence intensity recorded for both AL488 and TMR. In the case of TMR, a very large asymmetry exists in the effect of membrane potential on the fluorescence intensity. It can be speculated that TMR is largely within the reach of DPA (R_0_ = 35 Ǻ for TMR/DPA) at -50 mV and even more so at -150 mV. When a positive V_m_ is applied, the DPA that was located on the extracellular leaflet move toward the intracellular leaflet, a displacement of ~ 30 Ǻ. In this case, TMR is no longer within reach of DPA and the TMR fluorescence signal experiences a large increase. In comparison with TMR, the voltage-dependent change in AL488 fluorescence intensity is not as large because the larger R_0_ for the AL488/DPA pair (55 Å) makes that AL488 may always be within the range of some DPA molecules at all membrane potentials.

The next step was to assess the presence of FRET between AL488 and TMR (the calculated R_0_ for the AL488/TMR pair is 59Å) which would indicate that a significant fraction of C255A molecules are within molecular distances from each other as expected in the case of a multimeric complex. Fig 2C clearly show that in the presence of DPA and following V_m_ steps, control ocytes expressing wt hSGLT1 show little to no change in overall fluorescence (1.0 ± 0.4%, n = 5) using the FRET filter set. In sharp contrast, C255A expressing oocytes show a clear voltage-dependent FRET signal (9.0 ± 2.0%, n = 17) which is statistically different (p<0.05) from the signal obtained with wt hSGLT1. As depicted in Fig 1, this is what would be expected if SGLT1 was forming oligomers in the membrane.

**Fig 3:**
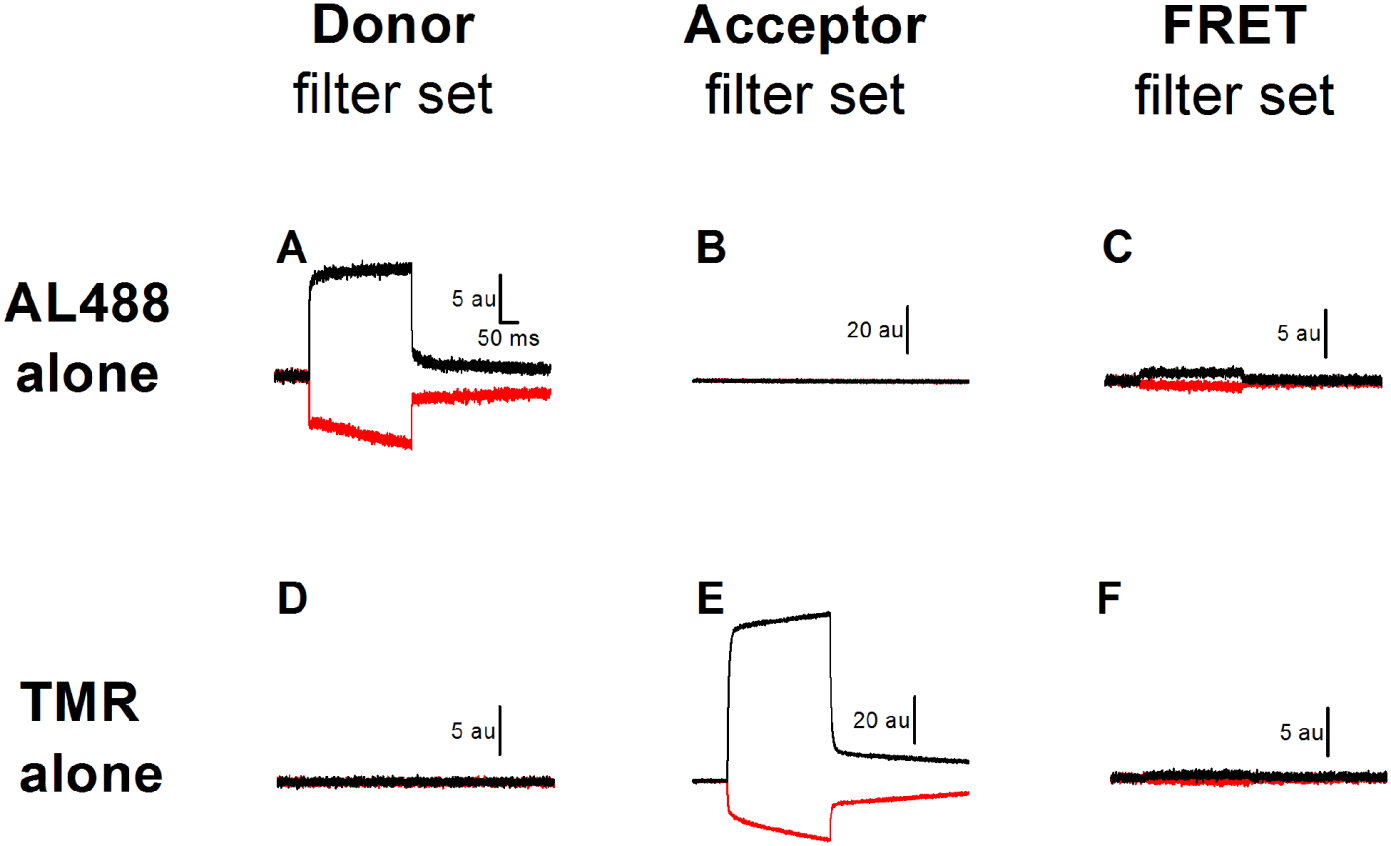
Control experiments performed in the absence of either the donor or the acceptor fluorophore. Typical fluorescence traces for oocytes expressing mutant C255A cotransporters in the presence of dipicrylamine (DPA) using 3 filter sets (Donor, Acceptor and FRET). When oocytes are only labelled with AL488 (panel A-C), membrane voltage pulses from -50 mV to +50 mV (black traces) and from -50 mV to -150 mV (red traces) produces a large change in the AL488 fluorescence signal which can be somewhat observed using the FRET filter set. The signal recorded with the FRET filter set (*F*_+50*mV*_ − *F*_−150*mV*_) averaged only 3.8 ±0.9% (mean ±SE, n = 6) of the signal directly measured with the Donor filter set. When C255A expressing oocytes are only exposed to the TMR (the acceptor) (panel D-F), the ratio between the FRET and the acceptor voltage-dependent fluorescence signals is 0.8 ±0.2% (mean ±SE, n = 5).

To solidly confirm that the voltage-sensitive FRET signal was really coming from the effect of DPA on a significant energy transfer mechanism, control experiments were performed where either the donor or the acceptor was missing (see Fig 3). First, let’s recall that, in the absence of DPA, oocytes expressing C255A do not generate any strong voltage-dependent fluorescent signal using either AL488 or TMR filter set (Fig 2D and 2E). Not surprisingly, under these conditions, there was no voltage-dependent signal with the FRET filter set (see Fig 2F for a representative example of 8 different experiments). When C255A expressing oocytes were labelled only with AL488 (the donor), a voltage-dependent fluorescence signal was present using the Donor filter set (Fig 3A) and a residual signal could be detected with the FRET filter set (Fig 3C). In average for 6 experiments, the FRET signal represents 3.8±0.9% (n = 6) of the signal detected with the Donor filter set. When C255A expressing oocytes were labelled only with TMR (the acceptor), a strong voltage-dependent fluorescence signal was present using the Acceptor filter set (Fig 3E) and a residual signal could be detected with the FRET filter set (Fig 3F) averaging 0.8 ± 0.2% (n = 5) of the signal detected with the Acceptor filter set. These two ratios were used to calculate the amplitude of the “FRET” signal that could be expected if AL488 and TMR were not exchanging any energy. Out of 14 experiments of the type shown in Fig 2 (panel G, H and I), the fluorescence level recorded with the FRET filter set was 3.0 ± 0.5-fold larger than the level predicted if there was no energy transfer (3.8% of the AL488 signal + 0.8% of the TMR signal). The difference between the observed and predicted fluorescence levels was highly significant (p<0.001). It is then concluded that the voltage-dependent FRET signal represents a true energy transfer between adjacent SGLT1 molecules labelled with AL488 and TMR.

### Western blotting

**Fig 4:**
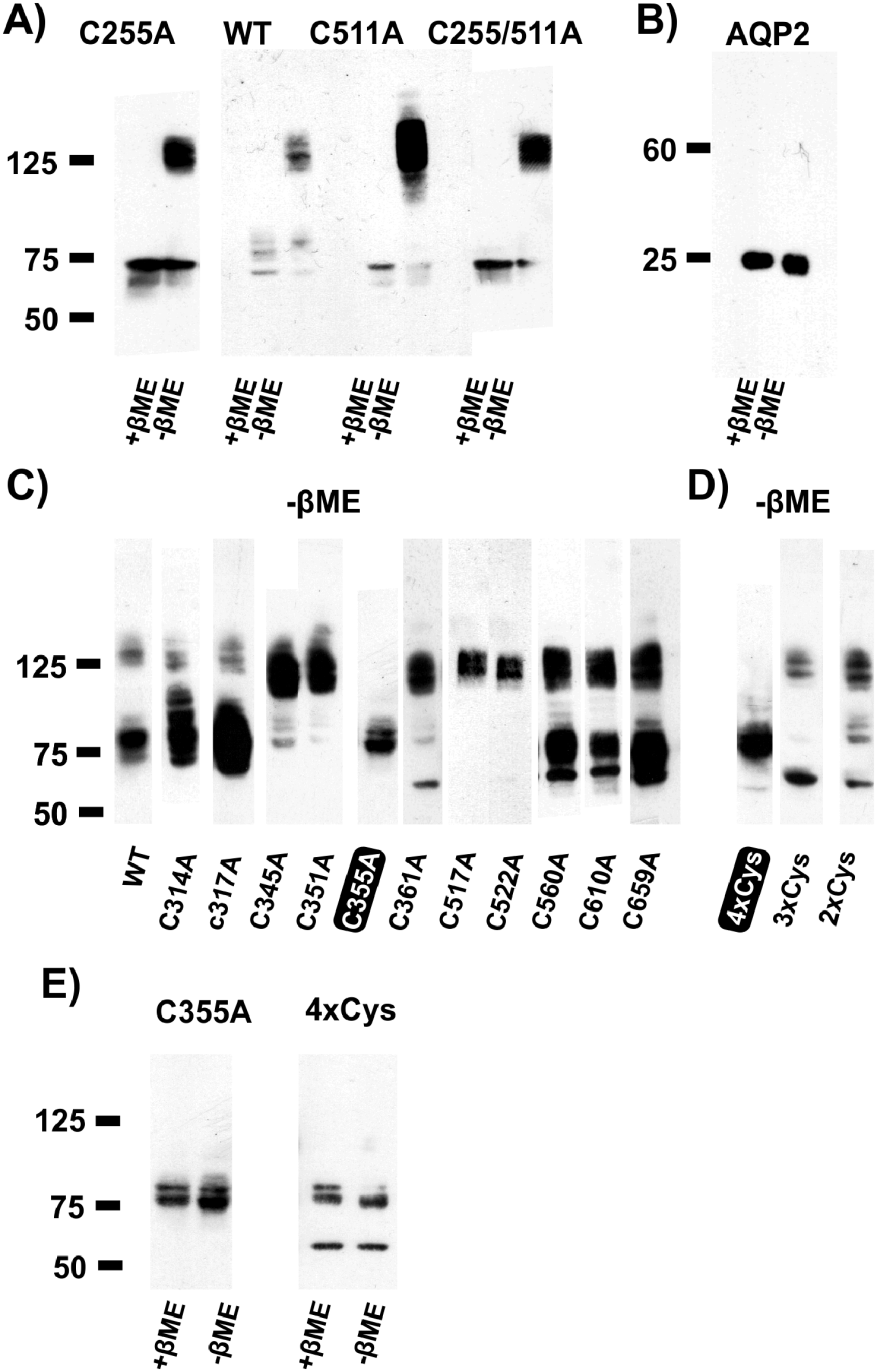
Western blot of wt and mutant myc—tagged hSGLT1. A) Effect of absence/presence of β-Mercaptoethanol (β-ME) on the migration profiles of the wt, the single cysteine mutants C255A and C511A and of the double cysteine mutant C255/511A. In the absence of β-ME, most of the cotransporters migrate at a level corresponding to a dimeric configuration. B) Absence of effect of β-ME on the migration profile of Aquaporin 2 (AQP2). C) Single cysteine mutants in the absence of β-ME. A band corresponding to a putative dimer is present for the wt and all tested single mutant, except C355A. D) Multiple mutants in absence of β-ME. A band corresponding to the putative dimer is present for 2xCys (C351/361A), and 3xCys (C345/351/361A), but not for 4xCys (C345/351/**355**/361A). E) β-ME has no effect on the migration profile for the mutants C355A and 4xCys. For all panels presented, experiments have been repeated 3 times and representative examples are shown.

While the hVoS FRET quenching experiments described above show that C255A can be found in a multimeric state, they do not allow any estimation of the multimeric level (dimer, trimer, tetramer…). The presence of a disulfide bridge was previously shown between C255 and C511 [3, 4]. Considering a homology model of hSGLT1 based on the structure of vSGLT, it is physically possible that this disulfide bridge could form between residues of different subunits. The present series of western blot experiments were performed to check for the presence of putative disulfide bridges linking neighboring subunits within a multimer. Western blotting of C255A, wt, C511A and C255/511A myc-tagged hSGLT1 was done in reducing and non-reducing conditions i.e. in the presence or absence of β-Mercaptoethanol (β-ME) (see Fig 4A. S1 Fig presents an uncropped version of Fig 4). As expected, wt hSGLT1 is detected at an apparent molecular weight of ~75 kDa in the presence of β-ME. In the absence of β-ME, hSGLT1 is detected at ~125 kDa which is consistent with a dimeric state stabilised by a disulfide bridge. As shown in Fig 4A, all related mutants (C255A, C511A and C255/511A) shared the migration pattern of wt hSGLT1 indicating that the β-ME-sensitive band observed at an apparent molecular weight of 125 kDa does not require a disulfide bridge that involves C255 or C511. As C511 is not required to observe a dimer in a western blot, this justifies *a posteriori* the use of C255A to investigate the multimeric state of hSGLT1 as done in Fig 2.

To check for the possibility that the absence of β-ME itself affected the migration pattern, we tested the effect of β-ME on the apparent molecular weight of aquaporin 2 (AQP2), a protein expressed as a tetramer without inter-subunit disulfide bridges. As expected, AQP2 was detected as a single band at 25 kDa with/without β-ME (see Fig 4B).

The next step was to identify the cysteine residue responsible for the crosslinking between the monomers. The effect of individual mutations of endogenous cysteines to alanines (i.e. C314A, C317A, C345A, C351A, C355A, C361A, C517A, C522A, C560A C610A and C659A) was assessed via western blotting in absence of β-ME. The remaining two cystein residues (C292 and C301) were not tested as, according to the structure of vSGLT, these residues would be embedded in the core of the cotransporter, and thus unlikely to have access to a partner cysteine on a different hSGLT1 molecule. Out of these 11 mutants, only C355A failed, in the absence of β-ME, to migrate to the level expected for a dimer (see Fig 4C).

Interestingly, four different cysteine residues (at position 345, 351, 355 and 361) are found on the 7–8 extracellular loop and have been the object of previous studies. The mutants C351A, C355A and C361A were found to be non-functional due to trafficking defects [4, 24]. Surprisingly, the double mutant C351/361A restored a correct cell-surface expression [24]. In addition, C355S is one of the mutants that produces the hereditary disease glucose-galactose malabsorption syndrome because of trafficking defects [25]. Taken together, these observations suggest that the 7–8 loop may be considered as a “hot spot” for disulfide bridge formation. Thus, the oligomeric state of a quadruple (4xCys: C345/351/355/361A), a triple (3xCys: 345/351/361A) and a double mutant (C351/361A) were tested. As shown in Fig 4D, only 4xCys (which is the only multiple mutant to contain the C355A mutation) did not produce any dimeric state in the absence of β-ME. Finally, Fig 4E confirms the detection of C355A and 4xCys as monomers both in presence and absence of β-ME. The fact that a single cysteine residue (C355) was identified as being responsible for the detection of a dimeric hSGLT1 in western blots suggests that the extracellular 7–8 loops of 2 neighbouring hSGLT1 molecules are linked together through a disulfide bridge between two C355 residues.

### hVoS quenching on E624C

We have previously shown that the SGLT1 mutant E624C could be labelled with a thiol-reactive fluorophore added to the extracellular solution [5]. As this position is within the 12–13 loop whose membrane localisation is debated, we intended to use DPA to solidly confirm that a cystein residue at position 624 is located on the extracellular side of the membrane.

Using oocytes expressing hSGLT1 as control for non-specific TMR labelling, adding DPA (at -50 mV) produced a decrease in TMR fluorescence intensity level by 6±1% (n = 9). In contrast, the effect of DPA on TMR fluorescence reached 12±3% (n = 8) using oocytes expressing E624C (p<0.05 vs wt hSGLT1). This suggests that TMR molecules attached to C624 can get closer to membrane bound DPA than non-specifically attached TMR molecules.

**Fig 5:**
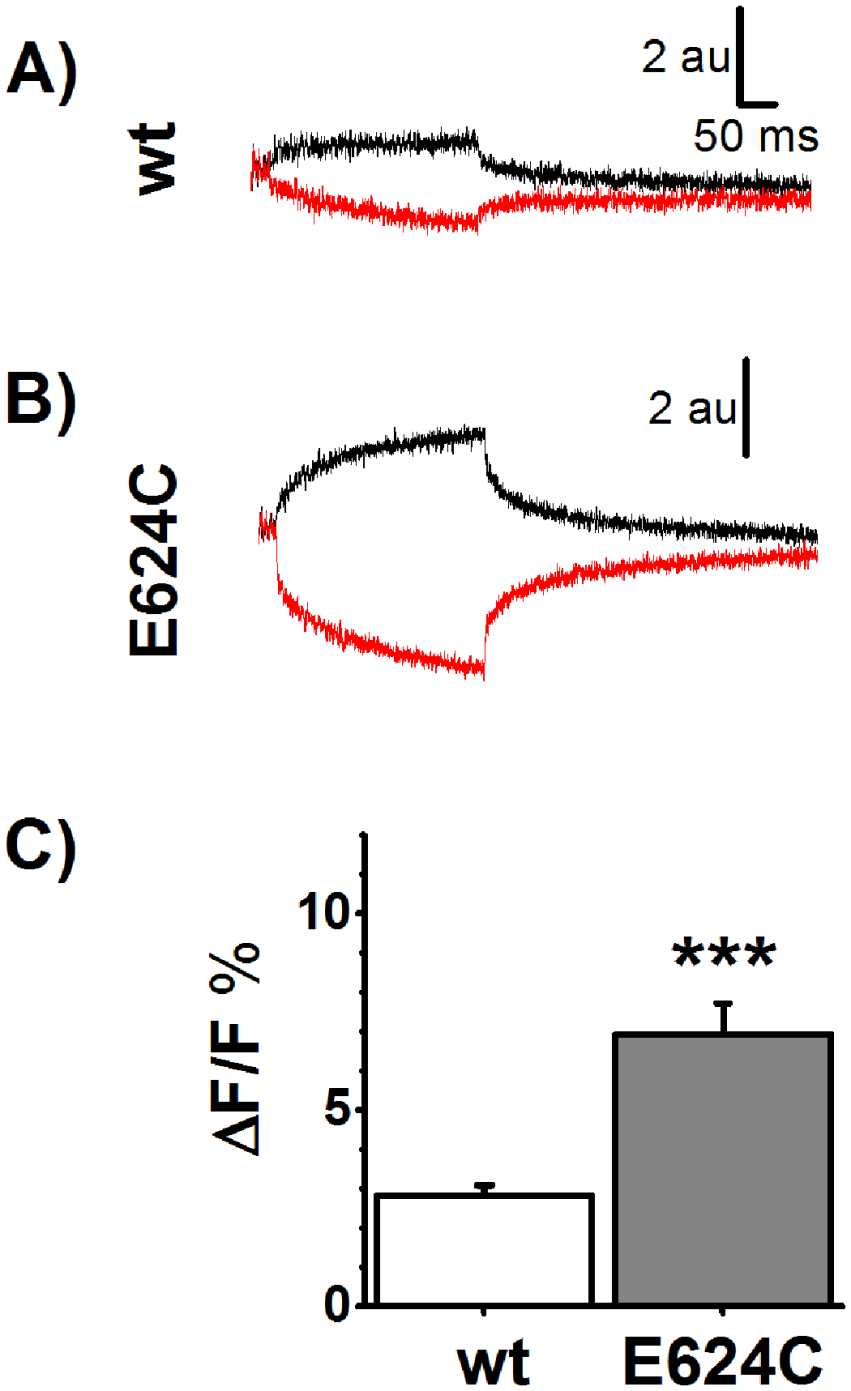
hVoS quenching on E624C. A) Typical fluorescence trace from wt hSGLT1 expressing oocytes labeled with Tetramethylrhodamine (TMR) in the presence of dipicrylamine (DPA). DPA is displaced by changing the membrane potential from -50 mV to +50 mV (black traces) and from -50 mV to -150 mV (red traces). B) Typical fluorescence trace from E624C expressing oocytes, labeled with TMR in the presence of DPA. C) Average voltage-sensitive fluorescence signal for wt hSGLT1 (mean±SE, n = 9) and for the mutant E624C (n = 8).*** denote statistical significance (p<0.001).

We then tested the effect of translocating DPA using a voltage step on the fluorescence level of oocytes expressing wtSGLT1 or E624C. Fig 5A and 5B present a representative example of experiments where the voltage-dependent fluorescence changes are clearly larger for E624C expressing oocytes than for hSGLT1 expressing oocytes. In average, going from -150 to +50 mV resulted in variations of the total fluorescence by 2.8±0.3% (n = 9) and 6.6±0.8% (n = 8) for wt hSGLT1 and E624C expressing oocytes, respectively (p<0.0001, see Fig 5C). The directionality of this voltage-dependent change in fluorescence intensity is consistent with a fluorophore located on the extracellular side of the membrane plane.

## Discussion

### hSGLT1 is expressed as a disulfide-bridged dimer

One of the main goals of this study was to determine the multimeric state of hSGLT1 in its natural membrane environment. While most of the literature so far assumed a monomeric cotransporter, effort to specifically address the question as yield several different results. Freeze-fracture studies [2] suggest a monomer configuration based on the observation of ~50 x 60 Å dots associated with the expression of hSGLT1. Crystallisation [12, 13] and some kinetic models [26, 27] would infer a dimer, and radiation inactivation studies on the proximal tubule Na/glucose cotransporter yield a target size of ~290 kDa that would be compatible with a tetramer [6]. As usual, each of these approaches can be criticized on its own right. Here, we used a two-step procedure to address hSGLT1 multimeric organisation. First, hVoS FRET quenching was used to show close vicinity between hSGLT1 molecules. Then, using western blotting under reducing or non-reducing conditions, we clearly observed that hSGLT1 migrates as a dimer held together by a disulfide bridge between the C355 residues of neighbouring subunits.

The AL488/TMR (donor-acceptor) pair used during hVoS FRET quenching provides a R_0_ of 59 Å. Based on the vSGLT crystal structure, this roughly correspond to the distance between the centers of two adjacent protomers regardless of the quaternary structure. This made this donor-acceptor pair perfectly suitable for the task at hand. While it was theoretically and technically feasible to only compare FRET signals recorded from hSGLT1 and C255A-expressing oocytes, use of DPA provides a number of advantages. During hVoS FRET quenching measurement, the variation of fluorescence following voltage steps is only related to the fluorophores that are in close proximity with the lipid leaflets where DPA sits (i.e. approximately within the AL488/DPA and TMR/DPA R_0_’s of 56 and 35 Å, respectively). This yields a significant improvement in the signal-to-noise ratio by greatly reducing the contribution of fluorophores that are non-specifically attached to oocytes (e.g. caught in membrane invaginations or in the vitelline layer that surrounds each oocyte). By eliminating a large part of the varying background fluorescence, DPA allows to separate the specific from nonspecific FRET signals. Another advantage of using DPA is that the direction of the voltage-dependent fluorescence signal provides direct information about the location of a fluorescent probe with respect to the membrane plane without requiring any other assumptions.

As shown in Fig 2 G to I, hVoS FRET quenching measurement unambiguously demonstrated that labeling of C511 by AL488 and TMR yielded a clear occurrence of FRET coming from fluorophores located on the extracellular side of the membrane. The voltage-dependent FRET signal is 5 to 7 times larger for C255A expressing oocytes than for wt SGLT1 expressing oocytes. This clearly indicates that a C255A protein labelled with AL488 has a significant chance of having a TMR-labelled partner within a distance of ~100 Å (for a distance larger than this limit, the probability of energy transfer is reduced to less than 4%). In the present study, we didn’t attempt to estimate the distance between AL488 and TMR with a better accuracy for 3 reasons: 1) in oocytes, a significant fraction of the fluorescence signal is non-specific and difficult to evaluate with precision, 2) the exact density of DPA in the membrane was not measured in each experiment, 3) the labelling efficacies reached with each of the two fluorophores are unknown. If usage of DPA gives a clear qualitative result (FRET or no FRET), the calculation of energy transfer between a donor and 2 acceptors requires several assumptions that defeat the purpose at the present time.

Once the “multimericity” of hSGLT1 has been established, we sought to observe the multimerisation level (i.e. dimer, trimer, tetramer) of hSGLT1. A promising approach was western blotting in presence and absence of β-ME. β-ME is a reducing agent specifically used during standard western blotting to break disulfide bonds. As shown in Fig 4A, in the presence of β-ME, hSGLT1 is only detected as a ~ 75 kDa monomer, consistent with previous findings [28]. In contrast, in absence of β-ME, wt hSGLT1 migrates mostly as a ~125 kDa protein which is consistent with a dimeric complex.

Mutants C255A, C511A and C255/511A were simultaneously tested. Exactly as with wt hSGLT1, the mutants produced a monomeric state with β-ME and a dimeric state when β-ME was omitted from the solution. This confirms the fact that C255A does behave as wt hSGLT1 and rules out the possibility of an artifactual multimerisation state caused by the use of C255A in hVoS FRET quenching experiments.

**Fig 6:**
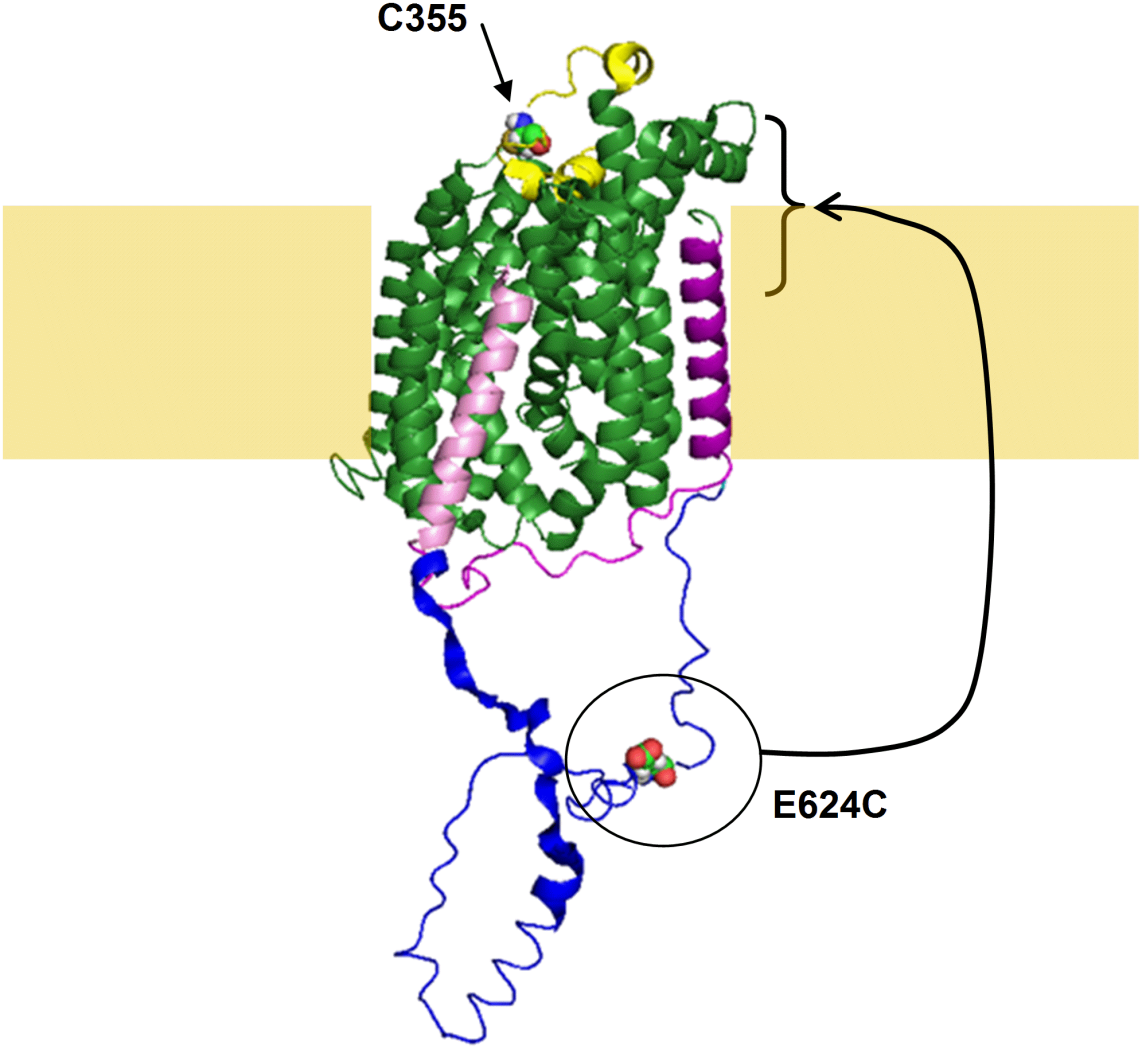
Expected location of C355 and comparison of the 12–13 loops from vSGLT and hSGLT1. A sequence alignment between vSGLT and hSGLT [12] showed that the position of C355 in hSGLT1 corresponds to N328 in vSGLT. Using vSGLT crystal structure (PDB# 3DH4) as a template, C355 would be located between TMS7 and 8, more precisely between extracellular helix 7a and 7b that are colored in yellow in the present figure (LeuT numbering of TMS’s). A top view shows that C355 would be located 10Ǻ away from the closest membrane-cotransporter interface. The figure also shows the difference in the loop linking TMS12 (in pink) and TMS13 (in purple). The 12–13 loop of vSGLT (in magenta) is 24 amino acids long and the corresponding loop in hSGLT1 (in blue) is 90 amino acids long. The position of E624 in the hSGLT1 12–13 loop is indicated. When mutated to a cysteine, that position can be labelled from the extracellular solution and hVoS FRET quenching suggested that this position should be located clearly above the membrane center.

We then start a search for the cysteine involved in this inter-subunit disulfide bridge. hSGLT1 contains a total of 15 cysteine residues. Out of these residues, C355 was unequivocally recognised as the cysteine residue responsible for the intermolecular disulfide bridge (see Fig 4C and 4D). Based on an aligment of the primary sequences of vSGLT and hSGLT1 [12], the position C355 would correspond to N328 in vSGLT. As shown in Fig 6, N328 is part of the extracellular 7–8 loop which is well conserved within the members of the SLC5A family. N318 (and, most likely, C355 in hSGLT1) is located at 10 Å from the closest edge of the cotransporter. A modest rearrangement of the 7–8 loop in hSGLT1 would easily allow it to form a disulfide bridge with the equivalent loop of a neighbouring subunit.

A number of roles can be attributed to the oligomeric states found in transporters and channels. In some cases, oligomerisation is essential as the transport pathway is formed by the interface between different protomers [29]. In cases where protomers are believed to be the basic functional unit, like in hSGLT1 [30], oligomerisation have been shown to be required for stability under stress condition [31], trafficking to the plasma membrane [32] and/or cooperativity/crosstalk during transport [33]. At the present time, it is difficult to speculate on the implication of hSGLT1 homodimeric state but, in a previous study [5], we have shown that breaking disulfide bridges with dithiothreitol (DTT) changes several kinetic parameters of hSGLT1. While the cotransporter Vmax is not affected by DTT, the affinity constant for alpha-methyl-glucose is doubled and the pre-steady state currents are significantly modified. In view of the present result, one of the possible explanations for this effect is that DTT disrupts the disulfide bridge that helps stabilising the dimeric state of the cotransporter.

### E624C is located on the extracellular side of the membrane

Accessibility and labeling of E624C in the 12–13 loop by TMR, a hydrophilic fluorescent probe added from the extracellular side, has been previously suggested by our group [5]. By using DPA, we aimed to unambiguously determine the position of this residue with respect to the membrane plane. As expected, the E624C mutation yielded a voltage-dependent fluorescent signal that is twice the signal that can be obtained with hSGLT1 (p<0.001). More interestingly, hyperpolarisation/depolarisation produced quenching/unquenching of TMR, respectively. This strongly confirms that TMR is located at least in the upper half of the lipid bilayer, i.e. on the external side. Furthermore, careful comparison of fluorescence time-courses from TMR-labeled C511 and E624C (Fig 2 Hand Fig 5B, respectively) reveal significant differences in shape. Explicitly, while fluorescent traces associated with the labeling of C511 showed the expected characteristic from a fluorophore located just above the membrane (i.e. large instantaneous quenching by DPA resulting from voltage steps followed by little to no variation) labeling of E624C by TMR showed a small instantaneous quenching by DPA upon application of a voltage pulse which is followed by a significant secondary variation. The low initial quenching/dequenching suggests that E624C needs to be located significantly above the membrane. The secondary displacements suggest that upon depolarisation (DPA moving inside) the amino acid at position 624 slowly move further away from DPA which decreases the level of quenching with time during the duration of the 300 ms pulse. Conversely, upon hyperpolarisation, the position 624 moves toward the membrane-bound DPA with time.

The intracellular localisation of the C-terminal of TM 12 is well accepted [5, 34]. In addition, it is in full agreement with the extracellular localisation of C511 (on 11–12 loop) which we confirmed in the present work. As part of 12–13 loop is located significantly above the membrane, we suggest that this loop has to be re-entrant. Such an assumption is structurally possible (see Fig 6). The 12–13 loop is composed of approximately 90 amino acids from K549 to K638 [5] and would be 135 Å in length if it was mostly alpha-helical.

Furthermore in contrast to the orientation of TM12, the position of the N-terminus of TM13 is still debated. SCAM accessibility study show extracellular accessibility of A664C (C-terminus of TM 13) based on labeling with both MTSES and MTSET [5], but other methods suggest intracellular localisation [35, 36]. While the crystal structure of vSGLT shows extracellular C-terminus [12, 13], lack of sequence homology between hSGLT1 and vSGLT and lack of structural homology between the different members of the LeuT structural family in this specific region prevent any definitive conclusions. Nevertheless, as the loop 12–13 is long enough to pass a 40 Å membrane twice, the possibility of a re-entrant loop is compatible with either orientations of TM 13.

A number of different roles have been associated with the 12–13 loop. It has been proposed to act as a extracellular binding site for sugar [37, 38] and inhibitors [39] [38–41], to be part of an extracellular vestibule [42], and to act as a intracellular binding site for sugars [43]. Those roles are not mutually exclusive. Comparison with other studies can give some insight on the possible conformation of loop 12–13. Wimmer *et al*. recently suggested that the early part of the 12–13 loop contained a non-stereospecific intracellular sugar binding site, while the later part contained a stereospecific extracellular glucose interaction site [43]. A study of the *E*. *coli* β-Glucoside Transporter BglF suggested that part of a cytoplasmic loop can alternate between inward- and outward-facing states [44]. Recent crystallisation of the glutamate-GABA antiporter GadC [45] show that the intracellular C-terminal fragment forms a folded domain, dubbed the C-plug. The C-plug completely blocks the path to the substrate binding site by inserting itself deep into the intracellular-facing putative substrate pathway. Finally, the 12–13 loop contains 37 charged amino acids (21 negatively charged and 16 positively charged) [5]. A trans-membrane configuration for this highly charged segment could allow it to play the role of the yet-to-be-identified SGLT’s voltage-sensor. Indeed, in the absence of substrate, SGLT1 can generate large transient current upon voltage pulse application that are reminiscent of gating currents in voltage-dependent channels [46]. The molecular origin of these currents has never been elucidated.

## Conclusions

Concomitant use of hVoS and FRET allowed us, without the need of extensive assumptions, to unambiguously show that C255A hSGLT1 is expressed as an oligomer at the oocyte membrane and that at least part of both the 11–12 loop (C511) and the 12–13 loop (E624C) are localised above the membrane plane. Western blotting further confirmed the oligomeric state, as removal of β-ME showed that wt hSGLT1 is expressed as a disulfide-bridged dimer. Further investigation showed that C355 is responsible for the disulfide bridge. Taken together, these results suggest that several differences are likely to exist between the actual structure of hSGLT1 and the crystal structure of vSGLT (quaternaly structure, position of 7–8 loop to allow an inter-molecular disulfide bridge and the re-entrant 12–13 loop). The proposed membrane configuration of the 12–13 loop could be involved in voltage sensing and could play a role in the way the cotransporter gets energized.

## Supporting Information

S1 Fig Uncropped version of the western blots presented in Fig 4. This figure presents the original western blots that were used to generate Fig 4.
(PNG)

## References

[1] Forrest LR, Krämer R, Ziegler C. The structural basis of secondary active transport mechanisms. Biochimica et Biophysica Acta (BBA)-Bioenergetics. 2010. 10.1016/j.bbabio.2010.10.014 21029721

[2] Eskandari S, Wright EM, Kreman M, Starace DM, Zampighi GA. Structural analysis of cloned plasma membrane proteins by freeze-fracture electron microscopy. Proceedings of the National Academy of Sciences. 1998;95(19):11235–40. 10.1073/pnas.95.19.11235 PMC21625 9736719

[3] Gagnon DG, Bissonnette P, Lapointe JY. Identification of a disulfide bridge linking the fourth and the seventh extracellular loops of the Na+/glucose cotransporter. The Journal of general physiology. 2006;127(2):145 16446504 10.1085/jgp.200509439 PMC2151483

[4] Gagnon DG, Frindel C, Lapointe JY. Voltage-Clamp Fluorometry in the Local Environment of the C255-C511 Disulfide Bridge of the Na+/Glucose Cotransporter. Biophysical journal. 2007;92(7):2403–11. 17208964 10.1529/biophysj.106.097964 PMC1864846

[5] Gagnon DG, Holt A, Bourgeois F, Wallendorff B, Coady MJ, Lapointe JY. Membrane topology of loop 13–14 of the Na+/glucose cotransporter (SGLT1): A SCAM and fluorescent labelling study. Biochimica et Biophysica Acta (BBA)-Biomembranes. 2005;1712(2):173–84. 15904891 10.1016/j.bbamem.2005.04.007

[6] Jetté M, Vachon V, Potier M, Béliveau R. Radiation-inactivation analysis of the oligomeric structure of the renal sodium/-glucose symporter. Biochimica et Biophysica Acta (BBA)-Biomembranes. 1997;1327(2):242–8. 9271266 10.1016/s0005-2736(97)00069-2

[7] Liu T, Speight P, Silverman M. Reanalysis of structure/function correlations in the region of transmembrane segments 4 and 5 of the rabbit sodium/glucose cotransporter. Biochemical and biophysical research communications. 2009;378(1):133–8. 10.1016/j.bbrc.2008.11.015 19013429

[8] Puntheeranurak T, Wildling L, Gruber HJ, Kinne RKH, Hinterdorfer P. Ligands on the string: single-molecule AFM studies on the interaction of antibodies and substrates with the Na+-glucose co-transporter SGLT1 in living cells. Journal of cell science. 2006;119(14):2960. 16787940 10.1242/jcs.03035

[9] Turk E, Kerner CJ, Lostao MP, Wright EM. Membrane topology of the human Na/glucose cotransporter SGLT1. Journal of Biological Chemistry. 1996;271(4):1925 8567640 10.1074/jbc.271.4.1925

[10] Longpré JP, Sasseville LJ, Lapointe JY. Simulated annealing reveals the kinetic activity of SGLT1, a member of the LeuT structural family. The Journal of general physiology. 2012;140(4):361–74. 23008432 10.1085/jgp.201210822 PMC3457693

[11] Wright EM, Loo DDF, Hirayama BA. Biology of human sodium glucose transporters. Physiological Reviews. 2011;91(2):733 10.1152/physrev.00055.2009 21527736

[12] Faham S, Watanabe A, Besserer GM, Cascio D, Specht A, Hirayama BA, et al The crystal structure of a sodium galactose transporter reveals mechanistic insights into Na+/sugar symport. Science. 2008;321(5890):810 10.1126/science.1160406 18599740 PMC3654663

[13] Watanabe A, Choe S, Chaptal V, Rosenberg JM, Wright EM, Grabe M, et al The mechanism of sodium and substrate release from the binding pocket of vSGLT. Nature. 2010;468(7326):988–91. 10.1038/nature09580 21131949 PMC3736980

[14] Vinothkumar KR, Raunser S, Jung H, Kühlbrandt W. Oligomeric structure of the carnitine transporter CaiT from Escherichia coli. Journal of Biological Chemistry. 2006;281(8):4795–801. 16365043 10.1074/jbc.M508993200

[15] Henry LK, DeFelice LJ, Blakely RD. Getting the message across: a recent transporter structure shows the way. Neuron. 2006;49(6):791–6. 16543127 10.1016/j.neuron.2006.03.002

[16] Tyagi NK, Puntheeranurak T, Raja M, Kumar A, Wimmer B, Neundlinger I, et al A biophysical glance at the outer surface of the membrane transporter SGLT1. Biochimica et Biophysica Acta (BBA)-Biomembranes. 2010. 10.1016/j.bbamem.2010.07.028 20692230

[17] Gagnon DG, Holt A, Bourgeois F, Wallendorff B, Coady MJ, Lapointe JY. Membrane topology of loop 13–14 of the Na+/glucose cotransporter (SGLT1): a SCAM and fluorescent labelling study. Biochim Biophys Acta. 2005;1712(2):173–84. Epub 2005/05/21. S0005-2736(05)00105-7 [pii] 10.1016/j.bbamem.2005.04.007 . 15904891

[18] Wang D, Zhang Z, Chanda B, Jackson MB. Improved Probes for Hybrid Voltage Sensor Imaging. Biophysical journal. 2010;99(7):2355–65. 10.1016/j.bpj.2010.07.037 20923671 PMC3042572

[19] Chanda B, Asamoah OK, Blunck R, Roux B, Bezanilla F. Gating charge displacement in voltage-gated ion channels involves limited transmembrane movement. NATURE-LONDON. 2005;7052:852. 10.1038/nature03888 16094369

[20] Groulx N, Juteau M, Blunck R. Rapid topology probing using fluorescence spectroscopy in planar lipid bilayer: the pore-forming mechanism of the toxin Cry1Aa of Bacillus thuringiensis. The Journal of general physiology. 2010;136(5):497 10.1085/jgp.200910347 20974771 PMC2964520

[21] Bissonnette P, Noël J, Coady MJ, Lapointe JY. Functional expression of tagged human Na+—glucose cotransporter in Xenopus laevis oocytes. The Journal of Physiology. 1999;520(2):359–71. 10523405 10.1111/j.1469-7793.1999.00359.x PMC2269588

[22] Batulan Z, Haddad GA, Blunck R. An intersubunit interaction between S4-S5 linker and S6 is responsible for the slow off-gating component in Shaker K+ channels. Journal of Biological Chemistry. 2010;285(18):14005 10.1074/jbc.M109.097717 20202932 PMC2859562

[23] Raja M, Kinne RKH. Structural Insights into Genetic Variants of Na+/Glucose Cotransporter SGLT1 Causing Glucose—Galactose Malabsorption: vSGLT as a Model Structure. Cell Biochemistry and Biophysics.1–8. 10.1007/s12013-012-9352-3 22383112

[24] Xia X, Wang G, Peng Y, Jen J. Cys351 and Cys361 of the Na+/glucose cotransporter are important for both function and cell-surface expression. Archives of biochemistry and biophysics. 2005;438(1):63–9. 15885653 10.1016/j.abb.2005.04.010

[25] Martin M, Lostao M, Turk E, Lam J, Kreman M, Wright E. Compound missense mutations in the sodium/D-glucose cotransporter result in trafficking defects. Gastroenterology. 1997;112(4):1206–12. 9098004 10.1016/s0016-5085(97)70132-x

[26] Chenu C, Berteloot A. Allosterism and Na++-d-glucose cotransport kinetics in rabbit jejunal vesicles: Compatibility with mixed positive and negative cooperativities in a homo-dimeric or tetrameric structure and experimental evidence for only one transport protein involved. Journal of Membrane Biology. 1993;132(2):95–113. 8496949 10.1007/BF00239000

[27] Koepsell H, Spangenberg J. Function and presumed molecular structure of Na+-D-glucose cotransport systems. Journal of Membrane Biology. 1994;138(1):1–11. 8189427 10.1007/BF00211064

[28] Leduc-Nadeau A, Lahjouji K, Bissonnette P, Lapointe JY, Bichet DG. Elaboration of a novel technique for purification of plasma membranes from Xenopus laevis oocytes. Am J Physiol Cell Physiol. 2007;292(3):C1132–6. Epub 2006/11/03. 00136.2006 [pii] 10.1152/ajpcell.00136.2006 . 17079335

[29] Jensen MØ, Jogini V, Borhani DW, Leffler AE, Dror RO, Shaw DE. Mechanism of Voltage Gating in Potassium Channels. Science. 2012;336(6078):229–33. 10.1126/science.1216533 22499946

[30] Kanner BI. Structural biology: It's not all in the family. Nature. 2008;454(7204):593–4. 10.1038/454593a 18668099

[31] Herz K, Rimon A, Jeschke G, Padan E. β-Sheet-dependent dimerization is essential for the stability of NhaA Na+/H+ antiporter. Journal of Biological Chemistry. 2009;284(10):6337–47. 10.1074/jbc.M807720200 19129192

[32] Farhan H, Freissmuth M, Sitte H. Oligomerization of neurotransmitter transporters: a ticket from the endoplasmic reticulum to the plasma membrane. Neurotransmitter Transporters. 2006:233–49. 10.1007/3-540-29784-7_12 16722239

[33] Gärtner RM, Perez C, Koshy C, Ziegler C. Role of bundle helices in a regulatory crosstalk in the trimeric betaine transporter BetP. Journal of Molecular Biology. 2011. 10.1016/j.jmb.2011.10.013 22024596

[34] Tyagi NK, Puntheeranurak T, Raja M, Kumar A, Wimmer B, Neundlinger I, et al A biophysical glance at the outer surface of the membrane transporter SGLT1. Biochimica et Biophysica Acta (BBA)-Biomembranes. 2011;1808(1):1–18. 20692230 10.1016/j.bbamem.2010.07.028

[35] Lin JT, Kormanec J, Homerova D, Kinne RKH. Probing transmembrane topology of the high-affinity sodium/glucose cotransporter (SGLT1) with histidine-tagged mutants. Journal of Membrane Biology. 1999;170(3):243–52. 10441667 10.1007/s002329900553

[36] Turner JR, Lencer WI, Carlson S, Madara JL. Carboxyl-terminal Vesicular Stomatitis Virus G Protein-tagged Intestinal Na-dependent Glucose Cotransporter (SGLT1). Journal of Biological Chemistry. 1996;271(13):7738–44. 8631815 10.1074/jbc.271.13.7738

[37] Raja MM, Kipp H, Kinne RKH. C-terminus loop 13 of Na+ glucose cotransporter SGLT1 contains a binding site for alkyl glucosides. Biochemistry. 2004;43(34):10944–51. 15323554 10.1021/bi049106n

[38] Tyagi NK, Kumar A, Goyal P, Pandey D, Siess W, Kinne RKH. D-Glucose-recognition and phlorizin-binding sites in human sodium/D-glucose cotransporter 1 (hSGLT1): a tryptophan scanning study. Biochemistry. 2007;46(47):13616–28. 17983207 10.1021/bi701193x

[39] Raja M, Kinne RK. Identification of phlorizin binding domains in sodium-glucose cotransporter family: SGLT1 as a unique model system. Biochimie. 2015;115:187–93. Epub 2015/06/19. S0300-9084(15)00174-1 [pii] 10.1016/j.biochi.2015.06.003 . 26086341

[40] Raja MM, Tyagi NK, Kinne RKH. Phlorizin recognition in a C-terminal fragment of SGLT1 studied by tryptophan scanning and affinity labeling. Journal of Biological Chemistry. 2003;278(49):49154–63. 12954647 10.1074/jbc.M306881200

[41] Xia X, Lin JT, Kinne RKH. Binding of phlorizin to the isolated C-terminal extramembranous loop of the Na+/glucose cotransporter assessed by intrinsic tryptophan fluorescence. Biochemistry. 2003;42(20):6115–20. 12755613 10.1021/bi020695b

[42] Puntheeranurak T, Kasch M, Xia X, Hinterdorfer P, Kinne RKH. Three surface subdomains form the vestibule of the Na+/glucose cotransporter SGLT1. Journal of Biological Chemistry. 2007;282(35):25222 17616521 10.1074/jbc.M704190200

[43] Wimmer B, Raja M, Hinterdorfer P, Gruber HJ, Kinne RKH. C-terminal loop 13 of Na+/glucose cotransporter 1 contains both stereospecific and non-stereospecific sugar interaction sites. Journal of Biological Chemistry. 2009;284(2):983–91. 10.1074/jbc.M805082200 19010790

[44] Yagur-Kroll S, Amster-Choder O. Dynamic membrane topology of the Escherichia coli β-glucoside transporter BglF. Journal of Biological Chemistry. 2005;280(19):19306–18. 15755739 10.1074/jbc.M410896200

[45] Ma D, Lu P, Yan C, Fan C, Yin P, Wang J, et al Structure and mechanism of a glutamate-GABA antiporter. Nature. 2012;483(7391):632–6. 10.1038/nature10917 22407317

[46] Parent L, Supplisson S, Loo DD, Wright EM. Electrogenic properties of the cloned Na+/glucose cotransporter: I. Voltage-clamp studies. J Membr Biol. 1992;125(1):49–62. Epub 1992/01/01. . 1542106 10.1007/BF00235797

