# Supplementary figures and images for: The Human Sodium-Glucose Cotransporter (hSGLT1) Is a Disulfide-Bridged Homodimer with a Re-Entrant C-Terminal Loop
Source: PLoS One. 2016 May 3;11(5):e0154589. doi: 10.1371/journal.pone.0154589 (PMC4854415; doi:10.1371/journal.pone.0154589)

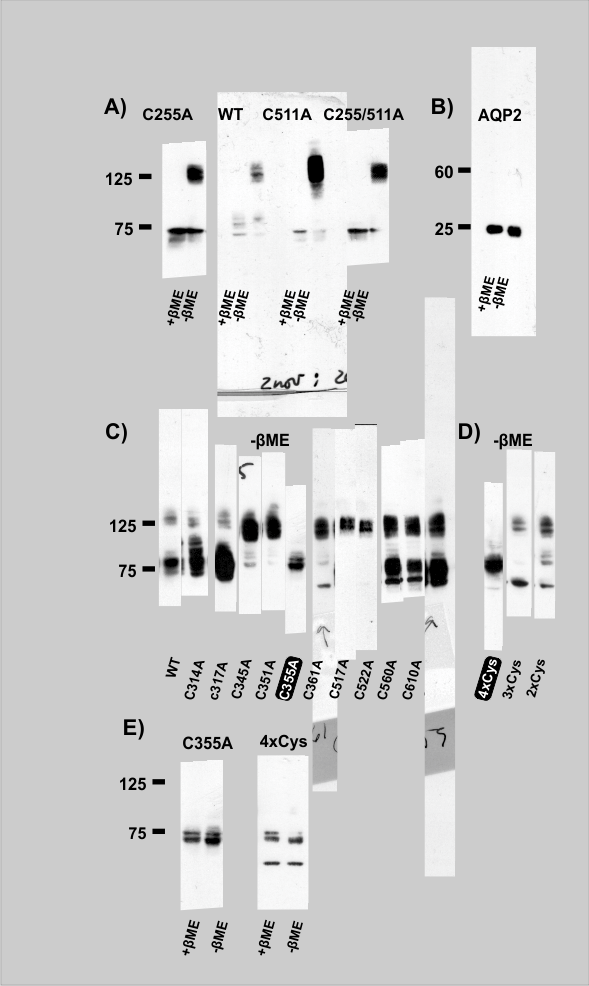

Supplement: S1 Fig — This figure presents the original western blots that were used to generate Fig 4. (PNG) [file pone.0154589.s001.png]
